# Diagnostic accuracy of a ‘stage-gated’ approach for reporting prostate screening MRI: “Is less more?”

**DOI:** 10.1007/s00330-025-12250-4

**Published:** 2026-02-19

**Authors:** Natasha Thorley, Tom Parry, Giorgio Brembilla, Francesco Giganti, Tristan Barrett, David Eldred-Evans, Nikhil Mayor, Alistair Lamb, Penny L. Hubbard Cristinacce, Fiona Gong, Henry H. Tam, Heminder K. Sokhi, Anwar R. Padhani, Caroline M. Moore, David Atkinson, Hashim U. Ahmed, Shonit Punwani

**Affiliations:** 1 Centre for Medical Imaging, University College London, London, UK; 2 Department of Radiology, University College London Hospital NHS Foundation Trust, London, UK; 3 Clinical and Experimental Radiology Unit, Experimental Imaging Center, IRCCS San Raffaele Scientific Institute, Milan, Italy; 4 Vita-Salute San Raffaele University, Milan, Italy; 5 Division of Surgical and Interventional Science, University College London, London, UK; 6 Department of Radiology, Addenbrooke’s Hospital and University of Cambridge, Cambridge, UK; 7 Division of Surgery, Imperial College London, London, UK; 8 Imperial Urology, Imperial College Healthcare NHS Trust, London, UK; 9 Department of Radiology, Imperial College Healthcare NHS Trust, London, UK; 10 Department of Radiology, The Hillingdon Hospitals NHS Foundation Trust, London, UK; 11 Paul Strickland Scanner Centre, Mount Vernon Hospital, Middlesex, UK; 12 Department of Urology, University College London Hospitals NHS Foundation Trust, London, UK

**Keywords:** Prostatic neoplasms, Mass screening, Magnetic resonance imaging, Prostate

## Abstract

**Objectives:**

To evaluate whether a two-step, ‘stage-gated’ reporting approach could improve the positive predictive value (PPV) of biparametric (bp)MRI for prostate cancer (PCa) screening compared to conventional Likert/PI-RADS scoring.

**Materials and methods:**

This retrospective secondary analysis utilised data from IP1-PROSTAGRAM—a prospective, population-based study of men aged 50–69 years who underwent PCa screening with bpMRI, ultrasound and prostate-specific antigen (PSA) testing between October 2018 and May 2019 at two centres (NCT03702439). MRI scans from IP1-PROSTAGRAM were retrospectively evaluated using the ‘stage-gated’ approach: three radiologists independently reviewed limited MRI sequences (axial T2-weighted and b1500 diffusion-weighted images) and classified scans as positive or negative; if positive, the remaining bpMRI images were reviewed and a hypothetical “decision-to-biopsy” made.

The PPV of ‘stage-gated’ reading was compared to PI-RADS and Likert scores ≥ 4 from the original IP1-PROSTAGRAM bpMRI reports. The reference standard was IP1-PROSTAGRAM biopsy results with grade group (GG) ≥ 2 cancer considered significant.

**Results:**

Of 408 participants (median age 57 years [IQR 53, 61]), 405 had MRI scans available for secondary analysis. The prevalence of GG ≥ 2 cancer was 4% (17/405). The ‘stage-gated’ reporting approach achieved a PPV of 53% (95% CI: 30, 75; 8/15), compared to 29% (95% CI: 15, 47; 8/28) and 30% (95% CI: 17, 46; 11/37) for Likert and PI-RADS ≥ 4 pathways, respectively. The ‘stage-gated’ approach halved the number of recommended biopsies while maintaining similar cancer detection rates.

**Conclusion:**

The ‘stage-gated’ reporting approach, using limited sequences for the initial read, may improve the PPV and benefit-to-harm ratio of MRI-based screening.

**Key Points:**

***Question**** The PPV of MRI in PCa screening is low, likely because conventional assessment systems are not optimised for low disease prevalence populations*.

***Findings**** A two-step, ‘stage-gated’ reading approach achieved a PPV of 53% (8/15), compared to 29% (8/28) for Likert and 30% (11/37) for PI-RADS scores ≥ 4*.

***Clinical relevance**** The ‘stage-gated’ reporting approach, which uses limited sequences for the initial read, may improve the PPV and benefit-to-harm ratio of MRI-based screening by reducing unnecessary biopsies. Prospective evaluation is needed to confirm these findings in real-world screening settings*.

**Graphical Abstract:**

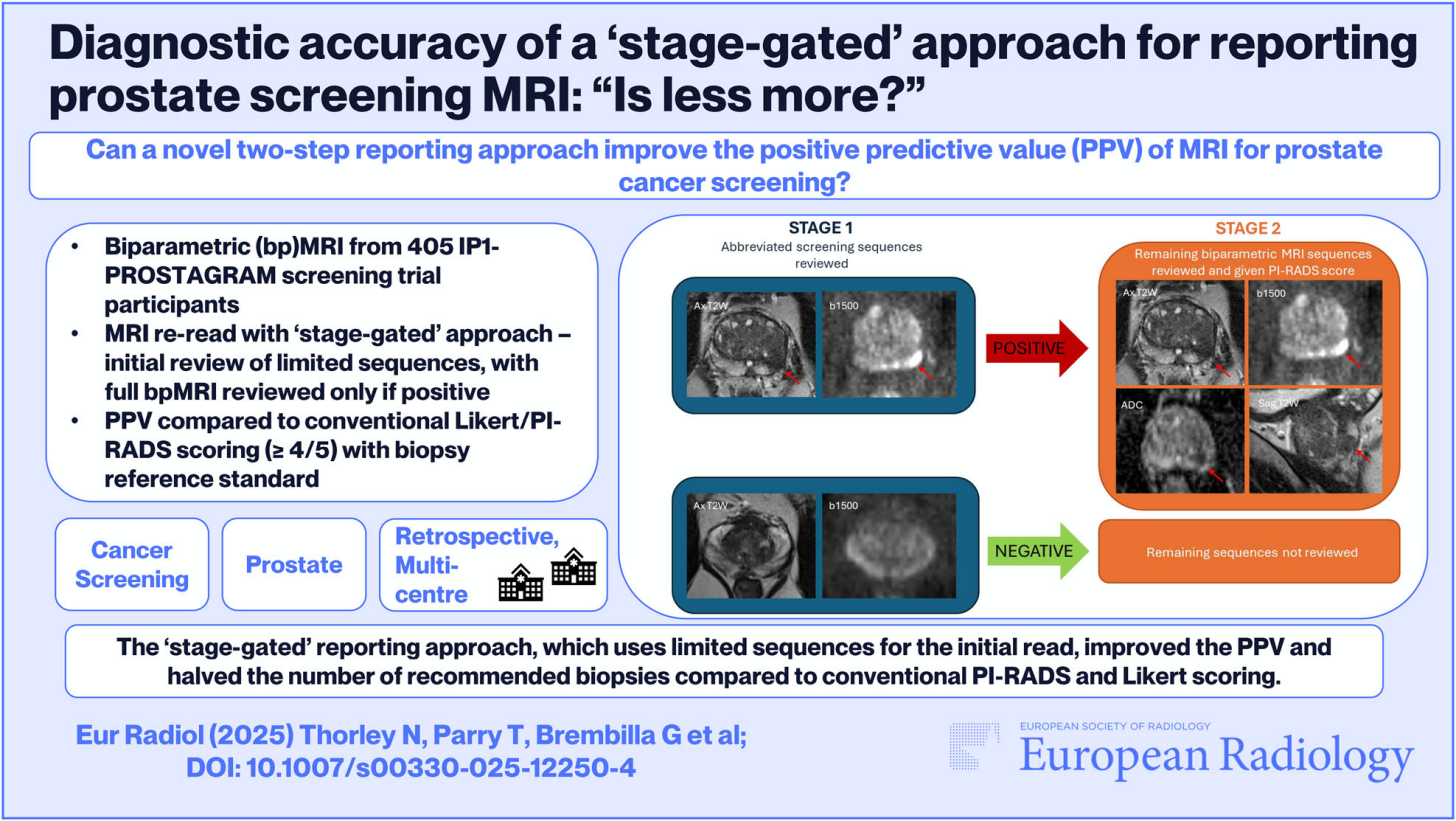

## Introduction

Prostate cancer (PCa) poses a substantial global health burden, and recent European Commission recommendations have urged further research into organised screening programmes [1]. A major challenge is establishing an approach that balances the benefits and harms of screening. Prostate-specific antigen (PSA) testing reduces mortality [2], but has notable limitations, including unnecessary biopsies and overdiagnosis [3, 4], leading to treatment-related harm.

Incorporating MRI into screening strategies may mitigate these harms by reducing unnecessary biopsies and enabling biopsy targeting, potentially improving the detection of clinically significant PCa [5–7]. Emerging evidence suggests shorter, non-contrast biparametric (bp)MRI protocols offer comparable effectiveness to multiparametric (mp)MRI [8–11], while improving feasibility for screening.

While MRI demonstrates high sensitivity for PCa detection, its positive predictive value (PPV) in a screening setting is relatively low. This is likely because conventional assessment systems, such as the Likert scale and prostate imaging reporting and data system (PI-RADS), were designed for mpMRI in higher-prevalence diagnostic settings and are not optimised for low-prevalence screening populations. For example, the PPV of Likert/PI-RADS scores of ≥ 4 on bpMRI in screening contexts ranges from 27% to 46% [12–15], leading to unnecessary biopsies in a significant proportion of men.

In the ReIMAGINE Screening Study [16, 17], abbreviated bpMRI using only axial T2-weighted imaging (T2WI) and high *b-*value (2000 s/mm²) diffusion-weighted imaging (DWI) at 3-T achieved a PPV of 59% (95% CI: 44, 72), rising to 86% (95% CI: 69, 95) when followed by a separate mpMRI in screen-positive cases [18]. This high PPV suggests that using a limited set of key sequences for the initial read may raise the threshold for positivity and better differentiate between positive and negative scans. While ReIMAGINE involved two separate MRI examinations, with mpMRI at the second stage, we aimed to improve practicality and efficiency for screening by simulating a similar stepwise strategy for reporting a single bpMRI examination. This involved first reviewing limited sequences and proceeding to review the remaining sequences only if the criteria for screen-positivity were met.

In this retrospective study, we utilised the IP1-PROSTAGRAM dataset [12, 19] to evaluate whether this two-step, ‘stage-gated’ reporting approach could improve the PPV of bpMRI for PCa screening compared to conventional Likert/PI-RADS scoring designed for mpMRI reading.

## Materials and methods

### Study population

This retrospective secondary analysis used data from the IP1-PROSTAGRAM study (NCT03702439), which received approval from the UK National Research Ethics Committee (8/LO/1338). All participants provided written informed consent. IP1-PROSTAGRAM protocol and outcomes have been previously published [12, 19].

IP1-PROSTAGRAM was a prospective, population-based PCa screening study that recruited men aged 50–69 years between October 2018 and May 2019. Eligible participants had a life expectancy of ≥ 10 years and none of the following exclusion criteria: history of PSA testing or prostate MRI within the past 2 years, recent urinary infection or prostatitis, prior prostate biopsy or PCa, or MRI contraindications (Supplementary Table S1).

### IP1-PROSTAGRAM study procedures

Participants underwent screening with PSA testing, MRI, and ultrasound.

BpMRI sequences included axial and sagittal T2WI, and DWI with multiple *b*-values (0, 150, 400, 1000, and 1500 s/mm^2^) to derive apparent diffusion coefficient (ADC) maps. Acquisition time was 15 min (acquisition parameters are in Supplementary Table S2). Scans were performed at two centres using one 1.5-T (Siemens Magnetom Aera; 17%) and one 3-T scanner (Siemens Magnetom Verio; 83%) with pelvic phased-array coils. Scans with poor image quality were repeated, and participants with susceptibility artefact on DWI due to rectal gas were offered a flatus tube. An antispasmodic agent was administered unless contraindicated.

MRI scans were interpreted by two expert uroradiologists, blinded to PSA and clinical information (apart from age), using PI-RADS v2 [20] (adapted for non-contrast bpMRI) and Likert scores. Ultrasound was independently scored using a validated 5-point system [21]. A screen-positive result was defined as an MRI or ultrasound score ≥ 3/5, or a PSA ≥ 3 ng/mL. A biopsy was recommended if any test was screen positive. Participants underwent transperineal, systematic, 12-core biopsies with additional image fusion–targeted biopsies of all MRI and ultrasound lesions.

### ‘Stage-Gated’ study procedures

**Fig. 1:**
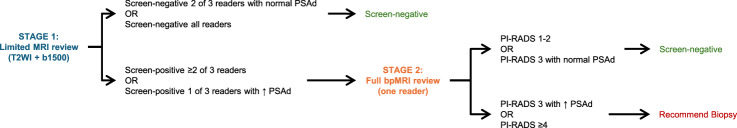
Schematic representation of the two-step ‘stage-gated’ reporting approach. *↑PSAd* raised prostate-specific antigen density (≥ 0.12 ng/mL^2^), *T2WI* T2-weighted imaging, *b1500* b1500 s/mm^2^ diffusion weighted imaging

In this secondary analysis, MRI scans from 405 participants were consecutively, retrospectively re-evaluated between August 2024 and December 2024 using a ‘stage-gated’ approach (Fig. 1).

#### Stage 1

Three expert uroradiologists (G.B., F.G., and T.B., with 8-, 11-, and 15-years’ prostate MRI experience, respectively), who were not involved in the original study, independently reviewed limited MRI sequences comprising only axial T2WI and b1500 DWI. Scans were classified as screen-positive, screen-negative, or non-diagnostic, based on prespecified criteria adapted from ReIMAGINE (Table 1) [18]. In brief, screen-positive scans required abnormal T2WI (score ≥ 3/5) with corresponding focal abnormality on b1500 DWI (score ≥ 4/5). Non-diagnostic scans had either (i) both sequences non-diagnostic, or (ii) one non-diagnostic sequence with a suspicious lesion (score ≥ 4/5) on the other. All other scans were screen-negative. Radiologists were blinded to all clinical and pathological data, including PSA, age, original screening classification, and histopathologic outcomes.

**Table 1:** Screening criteria used for stage 1 of the ‘stage-gated’ prostate MRI reporting approach, based on the criteria used in the ReIMAGINE screening study [18]

Axial T2	Axial b1500	Screen outcome
Focal 4–5/5 or diffuse 3/5	Focal 4–5/5	Positive
Focal 4–5/5	Focal 1–2/5 or diffuse 3/5	Negative
Diffuse 3/5	1–2/5 or diffuse 3/5	Negative
1–2/5	ANY	Negative
ANY	1–2/5	Negative
Non-diagnostic (artefact)	1–2/5 or diffuse 3/5	Negative
1–2/5 or diffuse 3/5	Non-diagnostic (artefact)	Negative
4–5/5	Non-diagnostic (artefact)	Non-diagnostic (recall)
Non-diagnostic (artefact)	4–5/5	Non-diagnostic (recall)
Non-diagnostic (artefact)	Non-diagnostic (artefact)	Non-diagnostic (no recall)

Lesions scoring 3/5 on T2 with a focal area that is slightly more intense than background diffuse change (but not qualifying as 4/5) are encompassed under diffuse 3/5

#### Advancement to Stage 2

Scans were advanced to Stage 2 if deemed screen-positive by at least two of three radiologists. In cases where only one radiologist classified a scan as screen-positive, the scan was advanced to Stage 2 if PSA density was elevated (PSAd ≥ 0.12 ng/mL^2^).

#### Stage 2

At this stage, the full bpMRI (including ADC maps and sagittal T2WI) was reviewed by one radiologist (G.B., F.G., or T.B.) who assigned a bpMRI-adapted PI-RADS v2.1 [22] score. The radiologist was blinded to the reason the scan was advanced to Stage 2. A retrospective, hypothetical biopsy recommendation was made for scans scoring PI-RADS ≥ 4, or PI-RADS 3 with elevated PSAd.

This stepwise approach balances specificity through reader consensus while preserving sensitivity by incorporating a PSAd threshold in discordant (Stage 1) or equivocal (Stage 2) cases. Although three readers reviewed scans concurrently, the outcomes are functionally equivalent to a two-reader workflow with a third reader acting as an arbitrator, and this design enabled assessment of alternative screening pathways. The PSAd threshold (≥ 0.12 ng/mL^2^) was selected based on its ability to identify clinically significant cancers missed on abbreviated MRI in ReIMAGINE [17, 18].

### Exploratory analyses

To assess the robustness of the ‘stage-gated’ approach, we performed sensitivity analyses exploring different combinations of readers at Stage 1 and criteria without the additional PSAd thresholds. These analyses aimed to evaluate whether modifying key pathway components would affect diagnostic accuracy. Full details are provided in the Supplementary Material.

### Outcome measures

The primary outcome was the PPV of the ‘stage-gated’ approach and bpMRI-adapted PI-RADS and Likert scores ≥ 4 from the original IP1-PROSTAGRAM MRI reports. Secondary outcomes included the number of recommended biopsies, detection of grade group (GG) ≥ 2 cancers, and benefit-to-harm ratios defined by Schoots et al [5]. Sensitivity, specificity, and negative predictive value are not reported due to the absence of reference standard verification in screen-negative participants.

The reference standard was IP1-PROSTAGRAM biopsy, with significant cancer defined as any length of GG ≥ 2 cancer (Gleason score ≥ 3 + 4) on systematic or targeted biopsy.

### Statistical analysis

No formal sample size calculation was performed as the study was designed as a secondary analysis.

Scans reported as non-diagnostic at Stage 1 were excluded from the analysis. Due to a high initial rate of non-diagnostic classifications, all non-diagnostic scans were re-read following reader retraining.

PPV was defined as the proportion of participants recommended for biopsy with GG ≥ 2 cancer. For the primary analysis, PPV was calculated in participants with reference standard verification (complete-case analysis) with Wilson 95% confidence intervals [23]. For the exploratory analyses, PPV was estimated using both complete-case analysis and methods to adjust for verification bias and missing reference standard data, including multiple imputation (standard and penalised logistic regression) as described by Day et al [24] (see Supplementary Material). Statistical analysis was performed by T.P. using Stata (v18.5, StataCorp).

## Results

### Characteristics

Of 408 consented IP1-PROSTAGRAM participants (median age 57 years [IQR 53, 61]), 405 had scans available for secondary analysis. Participant characteristics are shown in Table 2. The prevalence of GG ≥ 2 cancer was 4% (17/405).

**Table 2:** Characteristics of IP1-PROSTAGRAM participants

Characteristic	Participants *N* = 408
Age (years)	57 (53, 61)
First-degree relative with PCa	
Present	43 (11)
Absent	360 (88)
Missing	5 (1)
PSA (ng/mL)	0.92 (0.57, 1.80)
PSA density (ng/mL^2^)	0.04 (0.02, 0.06)
Prostate volume (mL)	28 (22, 35)

Data are *n* (%) or median (IQR)

*PSA* prostate-specific antigen

### Stage-gated reporting outcomes

Following review of limited MRI sequences in Stage 1, 30 of 405 (7%) scans met criteria for Stage 2 evaluation - 23 scans were deemed screen-positive by consensus of at least two radiologists, and 7 scans were screen-positive by one radiologist only with raised PSAd. An additional 49 of 405 (12%) were screen-positive by one radiologist only but had normal PSAd, therefore were not advanced to Stage 2. At Stage 1, 39 of 405 (10%) scans were initially classified as non-diagnostic by at least one radiologist. Following reader retraining, this decreased to 20 of 405 (5%), which were excluded from further analysis (Supplementary Fig. S1).

The number of screen-positive scans at Stage 1 varied between readers (range 14–72; Table S3). Overall agreement between all three readers was 82% (95% CI: 78, 86; 316/385).

**Fig. 2:**
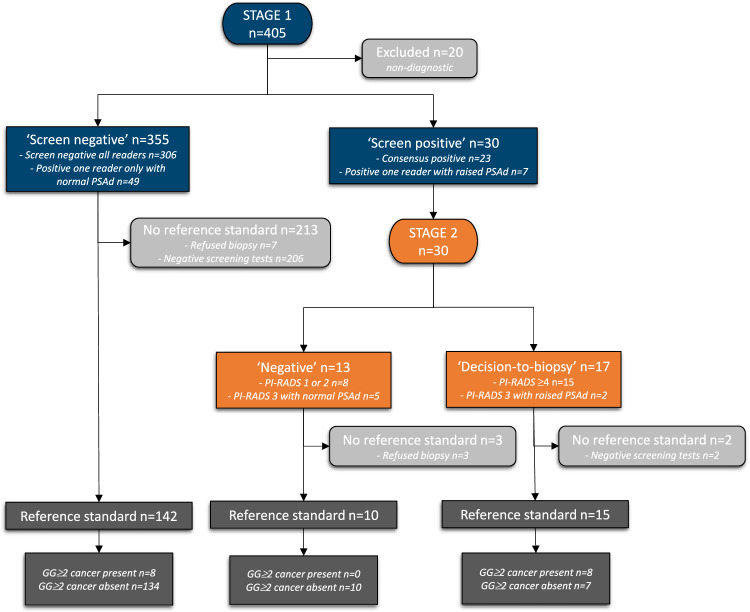
Flowchart of the ‘stage-gated’ pathway. *GG ≥ 2* grade group ≥ 2 cancer, i.e. clinically significant cancer, *PSAd* prostate-specific antigen density

Following full bpMRI evaluation in Stage 2, 17 of 30 (57%) men had a hypothetical biopsy recommendation—15 based on PI-RADS score ≥ 4 and 2 based on PI-RADS score 3 with raised PSAd. Of these men, 8 of 17 (47%) had GG ≥ 2 cancer, 1 of 17 (6%) had GG 1 cancer, 6 of 17 (35%) had no cancer, and 2 of 17 (12%) had no available reference standard outcome. Figure 2 shows the screening flowchart. The GG ≥ 2 to GG 1 ratio, which reflects the ability of the screening strategy to detect clinically significant cancer (GG ≥ 2) while minimising the detection of insignificant cancers (GG 1), was 8:1. The ‘stage-gated’ reporting approach demonstrated improved benefit-to-harm ratios [5] compared to both Likert and PI-RADS ≥ 4 pathways based on the original IP1-PROSTAGRAM scores (Table 3).

**Table 3:** Benefit-to-harm ratios [5] of the ‘stage-gated’ pathway compared to bp PI-RADS and Likert scores of ≥ 4 from the original IP1-PROSTAGRAM bpMRI reports

Outcome	Screening pathway (*N* = 385)		
	‘Stage-gated’ approach	bpPI-RADS ≥ 4	Likert ≥ 4
MRI-avoided biopsy	368	343	352
MRI-recommend biopsy	17	42	33
Biopsy outcome			
No biopsy	2 (12)	5 (12)	5 (15)
Benign	6 (35)	21 (50)	17 (52)
GG 1	1 (6)	5 (12)	3 (9)
GG ≥ 2	8 (47)	11 (26)	8 (24)
Benefit-to-harm ratios^5^			
GG ≥ 2/GG 1	8.0	2.2	2.7
GG ≥ 2/GG 1 + benign biopsy	1.1	0.4	0.4
Avoided biopsy/benign biopsy	61.3	16.3	20.7

The GG ≥ 2/GG 1 ratio reflects the ability of the screening strategy to selectively detect clinically significant cancers (GG ≥ 2), while minimising the detection of insignificant cancers (GG 1). The GG ≥ 2/GG 1 + benign biopsy ratio reflects the overall efficiency of the screening strategy in detecting clinically significant cancer (GG ≥ 2) relative to the number of insignificant (GG 1) or benign biopsies. The avoided biopsy/benign biopsy ratio reflects the balance of men avoiding biopsy (in those with negative MRI results) and benign biopsies (in those with positive MRI results)

Data are *n* or *n* (%)

*GG* grade group, *GG* *≥* *2* clinically significant cancer, *GG 1* clinically insignificant cancer

### Diagnostic accuracy

**Fig. 3:**
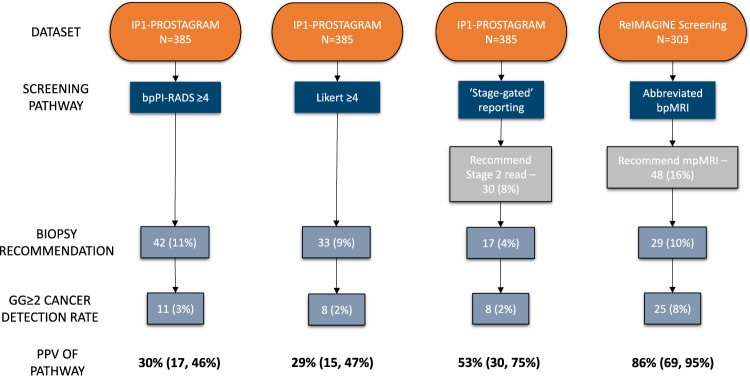
Diagnostic performance of the ‘stage-gated’ reporting approach compared to bpPI-RADS and Likert scores of ≥ 4 from the original IP1-PROSTAGRAM bpMRI reports. Diagnostic performance of the ReIMAGINE screening pathway [18] is included for comparison. PPV, positive predictive value; GG ≥ 2, grade group ≥ 2 cancer, i.e. clinically significant cancer. Data are *n* (%) or % (95% CI)

The PPV of the ‘stage-gated’ reporting pathway was 53% (95% CI: 30, 75; 8/15), compared to 29% (95% CI: 15, 47; 8/28) for the Likert ≥ 4, and 30% (95% CI: 17, 46; 11/37) for the PI-RADS ≥ 4 pathways. The ‘stage-gated’ approach recommended biopsy in approximately half as many men as the Likert ≥ 4 and PI-RADS ≥ 4 pathways (17 vs 33 and 42, respectively) (Fig. 3).

**Fig. 4:**
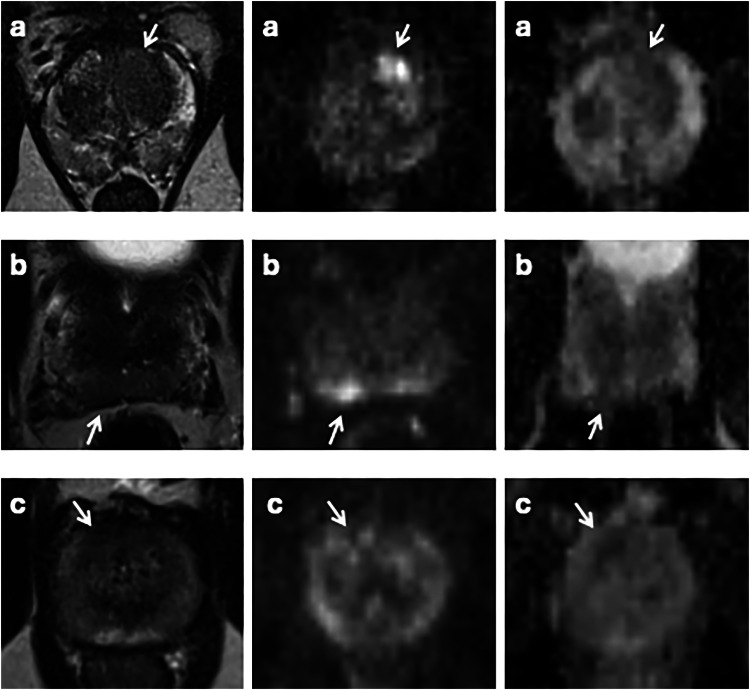
Three cases (**a**–**c**) of GG ≥ 2 PCa detected by the bpPI-RADS ≥ 4 pathway but missed by both the ‘stage-gated’ reading and Likert ≥ 4 pathways. The GG ≥ 2 lesion (arrow) is shown on axial T2WI (left), high *b*-value (b1500) DWI (middle), and ADC maps (right). Scans were all performed at 3-T. **a** A 63-year-old man with PSA 1.7 ng/mL and PSA density (PSAd) 0.05 ng/mL^2^. **b** A 58-year-old man with PSA 1.0 ng/mL and PSAd 0.03 ng/mL^2^. Both (**a**, **b**) were positive by only one radiologist in Stage 1. Due to normal PSAd, Stage 2 review was not performed. These cases were classified as radiologist interpretation errors. **c** A 59-year-old man with PSA 1.8 ng/mL and PSAd 0.05 ng/mL^2^. The screening images were negative by all radiologists in Stage 1, so they did not have Stage 2 review. In retrospect, the lesion is mainly conspicuous on the ADC map. This case was classified as a limitation of the reading strategy

Of 17 men with GG ≥ 2 cancer within the entire cohort, the PI-RADS ≥ 4 pathway detected 11 cases, while the ‘stage-gated’ and Likert ≥ 4 pathways both detected 8. The three GG ≥ 2 cancers identified by PI-RADS ≥ 4 but missed by the ‘stage-gated’ pathway did not advance to Stage 2 (two were screen-positive by only one radiologist with normal PSAd, and one was screen-negative by all radiologists) (Fig. 4). Of the remaining six men with GG ≥ 2 cancer, five were classified as screen-negative by all radiologists at Stage 1, and one was classified as non-diagnostic.

Among the men with PI-RADS or Likert scores ≥ 4 who would have correctly avoided biopsy by being classified as consensus screen-negative at Stage 1, in a considerable proportion (9/20, 45%), the elevated PI-RADS score was driven by appearances on the ADC map (Supplementary Table S4).

### Exploratory analyses

Using multiple imputation to account for missing reference standard data in the two men who would have received a biopsy recommendation on ‘stage-gated’ reading but were negative in the original study, the PPV of the ‘stage-gated’ pathway was 47% (95% CI: 26, 69).

The PPVs of alternative screening pathways are detailed in the Supplementary Material (Tables S6–S9). PPV was comparable for pathways with two readers with a third acting as an arbitrator (PPV 55%, 6/11, 95% CI: 28, 79); however, the inclusion of the PSAd threshold at Stage 1 detected two additional GG ≥ 2 cancers. In contrast, pathways relying on single-reader decisions showed greater variability in PPV.

## Discussion

The ‘stage-gated’ approach for bpMRI reading demonstrated a higher PPV than adapted Likert and PI-RADS scoring, achieving a PPV of 53% (95% CI: 30, 75), compared to 29% (95% CI: 15, 47) and 30% (95% CI: 17, 46) for the Likert and PI-RADS ≥ 4 pathways, respectively. This approach halved the number of recommended biopsies while maintaining similar cancer detection rates and improving benefit-to-harm ratios.

Minimising harm is a fundamental requirement of screening programmes, making the reduction of false positives and unnecessary biopsies critical to optimising the balance of benefits and harms. In line with recent PCa screening literature [5, 7], we focused on PPV and benefit-to-harm ratios as key diagnostic accuracy measures, as these can be robustly estimated in a screening context with biopsy verification.

The ‘stage-gated’ approach was designed to replicate the ReIMAGINE Screening [17] pathway using a single bpMRI examination. The PPV of ‘stage-gated’ reporting (53%) was lower than that of the overall ReIMAGINE pathway (86%, 95% CI: 69, 95), which incorporated mpMRI in screen-positive cases, and slightly lower than the abbreviated bpMRI (59%, 95% CI: 44, 72) [18]. This may reflect the lower prevalence of GG ≥ 2 cancers in our cohort (4% vs 10%), which directly impacts PPV, as well as the use of b1500 rather than b2000 DWI at 3-T. Nevertheless, the PPV of ‘stage-gated’ reading exceeded traditional scoring methods in IP1-PROSTAGRAM [12] and was higher than the PI-RADS ≥ 4 threshold reported in VISIONING [14] (28%, 95% CI: 19, 40) and MVP [13] screening studies (46%, 95% CI: 28, 65).

Among men with PI-RADS and Likert scores ≥ 4 who were correctly screened out at Stage 1, elevated scores were often driven by ADC findings with limited conspicuity on high *b*-value DWI, highlighting the importance of the high *b*-value DWI sequence [25]. In ‘stage-gated’ reading, a focal abnormality scoring ≥ 4/5 must be present on high *b*-value DWI to be considered screen-positive, which sets a higher threshold for positivity and reduces false positives. This may help to address the lower PPV of MRI in screening and improve the benefit-to-harm ratio by reducing unnecessary biopsies. A greater preponderance of GG ≥ 2 to GG 1 cancers may further reduce harm by improving histological selectivity.

The PI-RADS ≥ 4 pathway detected three additional GG ≥ 2 cancers compared to ‘stage-gated’ reporting. Two were likely due to radiologist interpretation error, and one was more conspicuous on the ADC map (a limitation of stage-gated reading). As this was a retrospective study, cases may have been more likely to be missed than in clinical practice, where reporting directly influences management. Other missed GG ≥ 2 cancers did not meet the MRI positivity threshold (no lesions scored ≥ 4/5). These missed cases are consistent with GÖTEBORG-2 [26] and STHLM3-MRI [27], where MRI served as a second-stage test following raised PSA. Repeated screening rounds may help mitigate the harms of missed cancers.

While incorporating MRI into the screening pathway may reduce the harms of PSA-based screening, MRI is costly and requires expert interpretation. ‘Stage-gated’ reading may improve reporting efficiency by allowing most scans to be assessed using only limited sequences, reducing reading time. Retrospective review of ReIMAGINE Screening and IP1-PROSTAGRAM datasets showed all serious incidental findings were visible on axial T2WI and high *b*-value DWI sequences, suggesting a low risk of harm from missed incidental pathology (Supplementary Table S10).

In our screening criteria, adapted from ReIMAGINE [18], scans with one non-diagnostic sequence were considered screen-negative if no suspicious lesion (≥ 4/5) was seen on the other sequence. If a suspicious lesion was present, the scan was classified as non-diagnostic, and the participant would be recalled. This approach differs from conventional reporting and requires specific training, as reflected by the re-read in our study. The higher threshold for a suspicious lesion on T2WI when b1500 DWI was non-diagnostic (≥ 4/5 vs ≥ 3/5 for screen-positive) was intended to minimise unnecessary recalls by reserving repeat imaging for higher-suspicion cases. In ReIMAGINE, half of the false-positive scans had equivocal (3/5) T2WI changes compared with only 15% of true-positives [18], underscoring the need to balance recall reduction with cancer detection. Our recall rate (5%) was higher than ReIMAGINE (< 1%) [18], though comparable screening data are lacking. In diagnostic settings, non-diagnostic (PI-QUAL < 3) rates of 5–11% have been reported [28, 29]. The lower recall rate in ReIMAGINE may reflect its use of a single 3-T scanner, highlighting the need for robust quality control in screening MRI implementation. Finally, scans were classified as non-diagnostic if any of the three readers reported them as such, reflecting a conservative approach. Further evaluation is needed to determine whether advancing such cases to Stage 2, when all sequences are available, could reduce recall rates.

Despite standardised reporting criteria, inter-reader variability was observed at Stage 1. While all readers were expert uroradiologists, tailored training for screening contexts—where disease prevalence differs from diagnostic populations—may improve inter-reader agreement. Although three readers reviewed scans concurrently, this configuration is functionally equivalent to a two-reader workflow with a third acting as an arbitrator, consistent with established double-reading models in population screening [30]. In clinical practice, a third reader would only be required in cases of disagreement; with 82% agreement between all three readers, this would be approximately 18% of cases. Two-reader pathways with a third as an arbitrator demonstrated comparable PPV, although the inclusion of the PSAd threshold at Stage 1 slightly improved GG ≥ 2 cancer detection. Further research could explore the role of artificial intelligence as a second reader, as is being investigated in breast cancer screening [31, 32].

This study has limitations. First, as a retrospective study, ‘stage-gated’ reporting did not influence biopsy decisions. While our findings demonstrate the feasibility of the approach and suggest a higher PPV over traditional scoring methods, prospective validation is needed. Data from the prospective ReIMAGINE study [18] support the potential success of such an approach. Second, screen-negative and some screen-positive men did not undergo disease status verification, introducing potential verification bias. Accordingly, we report biopsy-verified measures (e.g., PPV), which can be robustly estimated in a screening context. To address potential bias from missing reference standard data, complementary multiple imputation analyses were performed. This accounted for missing outcomes, including two men recommended for biopsy on ‘stage-gated’ reading but negative in the original study, and yielded consistent PPV estimates. Lastly, the small number of GG ≥ 2 cancers (4%) in this screening cohort led to wide confidence intervals, limiting statistical precision and potentially reducing the generalisability of our findings despite observed PPV improvements. Consequently, the study was not powered to formally test non-inferiority, and comparisons should be interpreted descriptively.

In conclusion, the ‘stage-gated’ approach for reporting prostate screening MRI demonstrated higher PPV than conventional Likert/PI-RADS scoring, halving unnecessary biopsies while maintaining comparable cancer detection. These findings suggest that using limited MRI sequences for the initial read may improve the benefit-to-harm ratio of MRI-based screening, providing supportive data for possible implementations in cancer screening trials.

## Supplementary information

ELECTRONIC SUPPLEMENTARY MATERIAL

## Abbreviations

ADC: Apparent diffusion coefficient
bpMRI: Biparametric MRI
DWI: Diffusion-weighted imaging
GG: Grade group
mpMRI: Multiparametric MRI
PCa: Prostate cancer
PPV: Positive predictive value
PSA: Prostate-specific antigen
PSAd: Prostate-specific antigen density
T2WI: T2-weighted imaging
