# Supplementary material for: Diagnostic accuracy of a ‘stage-gated’ approach for reporting prostate screening MRI: “Is less more?”
Source: Eur Radiol. 2026 Feb 19;36(7):5678–87. doi: 10.1007/s00330-025-12250-4 (PMC13282289; doi:10.1007/s00330-025-12250-4)
Supplement: Supplementary file 1 — ELECTRONIC SUPPLEMENTARY MATERIAL [file 330_2025_12250_MOESM1_ESM.pdf]

Diagnostic Accuracy of a ‘Stage-Gated’ Approach for Reporting Prostate Screening MRI: “Is Less More?”

ELECTRONIC SUPPLEMENTARY MATERIAL

Table S1 – IP1-PROSTAGRAM[1] study inclusion and exclusion criteria.

**Inclusion Criteria**
1. Men aged between 50 and 69 years inclusive at the time of study entry<br>2. Participants must be fit to undergo all procedures listed in the protocol<br>3. Estimated life expectancy of 10 years or more<br>4. An understanding of the English language sufficient to understand written and verbal information about the trial and consent process<br>5. Participants must be willing and able to provide written informed consent
**Exclusion Criteria**
1. Previous PSA test or prostate MRI within the prior two years of screening/consent visit<br>2. Evidence of a urinary tract infection or history of acute prostatitis within the last 6 months<br>3. Previous history of prostate cancer, prostate biopsy or treatment for prostate cancer (interventions for benign prostatic hyperplasia/bladder outflow obstruction is acceptable)<br>4. Any potential contraindication to MRI, including but not limited to:<br>a. Devices or metallic foreign bodies such as pacemakers, implantable defibrillators, neurostimulators, cochlear implants, coronary stents, prosthetic heart valves, aneurysm clips and other intravascular devices<br>b. Previous history of hip replacement surgery, metallic hip replacement or extensive pelvic orthopaedic metal<br>c. Claustrophobia<br>5. Any potential contraindication to prostate biopsy<br>6. Dementia or altered mental status that would prohibit the understanding or rendering of informed consent<br>7. Any other medical condition precluding procedures described in the protocol

Table S2 – IP1-PROSTAGRAM study MRI acquisition protocol[1]. Scans with poor quality images were repeated and if the quality of the diffusion-weighted imaging sequences was compromised by air, participants were offered a rectal flatus tube to decompress the rectum. To reduce motion artefact from bowel peristalsis, an antispasmodic agent was administered to all participants.

Sequence	Plane	TR (ms)	TE (ms)	Aver-ages	FA (degree)	WFS (pix)	BW (Hz/Px)	FoV (mm)	Phase FOV (% of FOV)	Over-sampling (% of FOV)	Phase enc. direction	Slice thickness (mm)	Slice gap (% of slice thickness)	TSE/EPI factor	FS method	Matrix	Phase res. (% of matrix)	Recon. voxel size (mm)	Sequence duration (mm:ss)
**3T SIEMENS MAGNETOM Verio syngo MR B17**																			
**Localiser**	Multiplanar	1000	92	1	150	1.2	349	400	100	20	Multiple	7	100	256		256	100	1.6x1.6x7	00:15
**T2 TSE**	Sagittal	7000	101	3	Min 150	2.0	200	200	100	43	H>F	3	20	25		320	80	0.8x0.6x3	02:57
**T2 TSE**	Axial	7000	108	2	Min 150	1.1	200	363	100	100	R>L	3	0	24		320	80	0.8x0.6x3	02:43
**DWI** (b0, 150, 400, 1000)	Axial	8500	80	3		0.2	1698	250	100	30	A>P	3	0	128	SPAIR	128	100	2 x 2 x 3	04:42
**DWI (b1500)**	Axial	9100	85	7		0.2	1698	250	100	30	A>P	3	0	128	SPAIR	128	100	2 x 2 x 3	03:40
**1.5T SIEMENS MAGNETOM Aera**																			
**Localiser**	Multiplanar	1000	93	1	180	0.4	501	400	100	20	Multiple	7	100	256		256	100	1.6x1.6x7	00:11
**T2 TSE**	Sagittal	5280	125	3	Min 150	1	200	200	100	100	H>F	3	20	23		320	80	0.6x0.6x3	03:17
**T2 TSE**	Axial	4590	135	3	Min 150	1	200	200	100	100	R>L	3	0	23		320	80	0.6x0.6x3	02:51
**DWI (b0, 150, 400, 1000)**	Axial	7500	67	2, 3, 4, 5		0.1	1507	250	100	30	A>P	3	0	128	SPAIR	128	100	2 x 2 x 3	05:23
**DWI (b1500)**	Axial	7500	68	9		0.1	1502	250	100	30	A>P	3	0	128	SPAIR	128	100	2 x 2 x 3	04:00
All scans were performed with intravenous administration of 20 mg hyoscine butylbromide. If contraindicated, 1 mg of glucagon hydrochloride was used intravenously. If both bowel relaxants were contra-indicated, no medication was used																			

Table S3 - Stage 1 outcomes by reader.

Reader	Stage 1 Outcome		
	Screen-positive	Screen-negative	Non-diagnostic
A	14	376	15
B	72	328	5
C	31	368	6

N = 405 total cases

Table S4 – Imaging features of scans with PI-RADS or Likert scores ≥4 on IP1-PROSTAGRAM which were correctly classified as screen-negative by consensus at Stage 1 of the 'stage-gated' approach. Imaging features were documented following retrospective analysis by an experienced uroradiologist (S.P.).

**Imaging Feature**	**Number of Scans N=20**
Elevated PI-RADS score based primarily on low ADC signal with limited conspicuity on high *b*-value DWI[a] – a limitation of the conventional scoring system	9
Radiologist overcall	6
Equivocal – lesion not well characterised on 'stage-gated' approach or conventional scoring	4
Artefact	1

ADC = apparent diffusion coefficient; DWI = diffusion-weighted imaging
[a]In some lesions with very low T2 signal (e.g. dense fibrosis, calcification), baseline signal intensity may be reduced, resulting in low ADC values even if the high *b*-value DWI does not appear hyperintense.

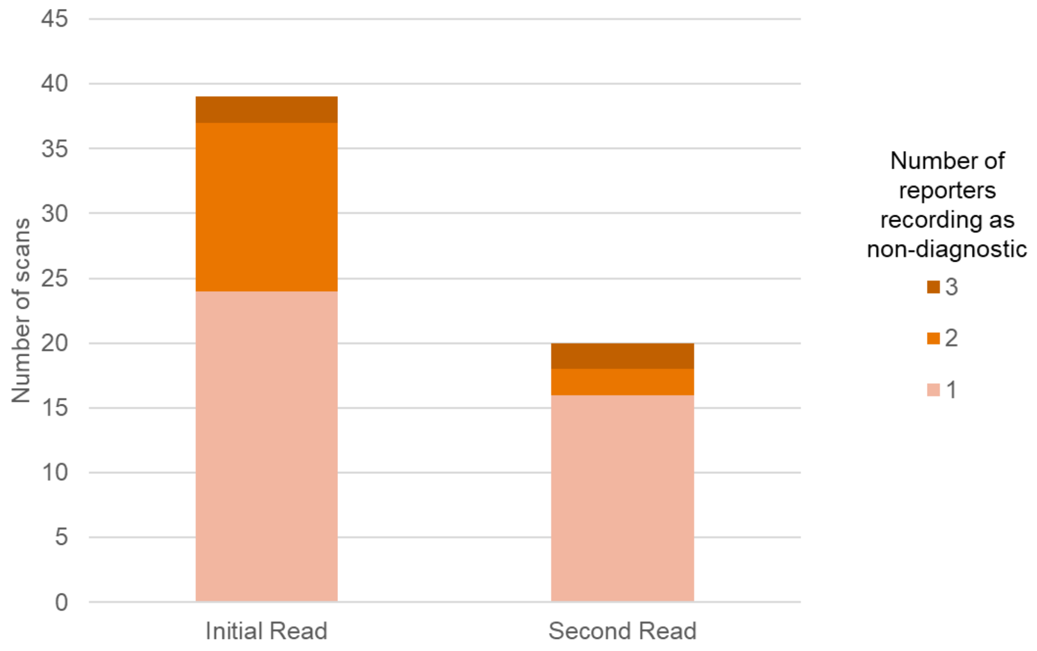

Figure S1 – Scans classified as non-diagnostic on the initial and second read. Due to a high proportion of non-diagnostic classifications on the initial read, all non-diagnostic scans were re-reviewed by all radiologists to ensure consistent application of the reporting criteria. Prior to the second read, the screening criteria were re-iterated: if either T2WI or DWI was non-diagnostic, the scan was to be classified as screen-negative unless a suspicious lesion (score ≥4/5) was visible on the other sequence.

Exploratory Analyses

Exploratory sensitivity analyses were conducted to evaluate the impact of modifying key criteria of the 'stage-gated' pathway on diagnostic accuracy (as measured by PPV). In total, 17 different screening pathways were compared as described in Table S5. These can be grouped in relation to the original 'stage-gated' pathway (pathway 1), and include pathways without PSAd thresholds as an additional screening criteria (pathways 2-4) and pathways with varying reader combinations at Stage 1 (pathways 5-17) - including three-reader, two-reader, and single-reader pathways.

Table S5 – descriptions of the 17 different screening pathways, including the original 'stage-gated' pathway (pathway 1). For each pathway, the criteria for advancement to Stage 2 and criteria for the hypothetical biopsy recommendation are listed.

Pathway	Description	Criteria for advancement to Stage 2	Criteria for biopsy recommendation
1	Original 'stage-gated' pathway	Screen-positive ≥2 of 3 readers or one reader with ↑ PSAd	PI-RADS ≥4 OR PI-RADS 3 with ↑ PSAd
2	No PSAd threshold at Stage 1 or 2	Screen-positive ≥2 of 3 readers	PI-RADS ≥4
3	No PSAd threshold at Stage 1	Screen-positive ≥2 of 3 readers	PI-RADS ≥4 OR PI-RADS 3 with ↑ PSAd
4	No PSAd threshold at Stage 2	Screen-positive ≥2 of 3 readers or one reader with ↑ PSAd	PI-RADS ≥4
5	Two readers with third as arbitrator	Screen-positive by reader A and B, with reader C as arbitrator if disagreement	PI-RADS ≥4 OR PI-RADS 3 with ↑ PSAd
6	Two readers with third as arbitrator	Screen-positive by reader A and C, with reader B as arbitrator if disagreement	PI-RADS ≥4 OR PI-RADS 3 with ↑ PSAd
7	Two readers with third as arbitrator	Screen-positive by reader B and C, with reader A as arbitrator if disagreement	PI-RADS ≥4 OR PI-RADS 3 with ↑ PSAd
8	Any one of three readers	Screen-positive by any one reader	PI-RADS ≥4 OR PI-RADS 3 with ↑ PSAd
9	Two-reader consensus	Screen-positive by reader A and B	PI-RADS ≥4 OR PI-RADS 3 with ↑ PSAd
10	Two-reader consensus	Screen-positive by reader A and C	PI-RADS ≥4 OR PI-RADS 3 with ↑ PSAd
11	Two-reader consensus	Screen-positive by reader B and C	PI-RADS ≥4 OR PI-RADS 3 with ↑ PSAd
12	One of two readers	Screen-positive by reader A or B	PI-RADS ≥4 OR PI-RADS 3 with ↑ PSAd
13	One of two readers	Screen-positive by reader A or C	PI-RADS ≥4 OR PI-RADS 3 with ↑ PSAd
14	One of two readers	Screen-positive by reader B or C	PI-RADS ≥4 OR PI-RADS 3 with ↑ PSAd

15	Single reader	Screen-positive by reader A	PI-RADS ≥4 OR<br>PI-RADS 3 with ↑ PSAd
16	Single reader	Screen-positive by reader B	PI-RADS ≥4 OR<br>PI-RADS 3 with ↑ PSAd
17	Single reader	Screen-positive by reader C	PI-RADS ≥4 OR<br>PI-RADS 3 with ↑ PSAd
↑ PSAd = raised prostate-specific antigen density (≥0.12 ng/mL^2)			

In screening studies, such as IP1-PROSTAGRAM, only participants with positive screening tests undergo verification of the target condition by the reference standard – in other words, only participants with positive screening tests undergo prostate biopsy. This introduces verification bias, and several statistical methods have been proposed to correct for this. One such method, the multiple imputation method, has been previously applied to the IP1-PROSTAGRAM dataset by Day et al[2]. This treats the condition status of the non-verified participants as a missing data problem and imputes the missing condition statuses[2].

In addition to missing data from absent reference standard outcomes, screening pathway 8 and 12-17, resulted in more scans being recommended for Stage 2 review compared to the original 'stage-gated' pathway. This meant that some participants had missing hypothetical biopsy decisions as this was made at Stage 2. For the purposes of analysis, this was treated as a missing data problem, similar to missing reference standard outcomes.

Several methods, including methods to adjust for verification bias and missing data, were used to calculate the PPV of the different screening pathways:

- **Complete case analysis** (unadjusted approach) (Table S6) – only participants with known Stage 2 outcome and reference standard outcomes were included. Participants with unknown Stage 2 outcome and non-verified condition status were omitted.
- **Minimum and maximum PPV estimates** (Table S7) – participants with missing outcome at Stage 2 were assumed to be recommended for biopsy. To calculate the minimum PPV, participants with missing reference standard outcomes were assumed to be false positive and to calculate the maximum PPV, participants with missing reference standard outcomes were assumed to be true positive (Figure S2).
- **Multiple imputation** (Table S8) – missing reference standard outcomes were imputed based on the original results of the three IP1-PROSTAGRAM screening tests (MRI, ultrasound, PSA) and the verified condition status, as per the method described by Day et al[2]. Due to the small number of participants with clinically significant cancer, Firth's correction[3] was applied alongside standard logistic

regression, to reduce small-sample size bias. Where Stage 2 outcome was missing, two PPV calculations were performed, assuming participants were and were not recommended for biopsy, respectively.

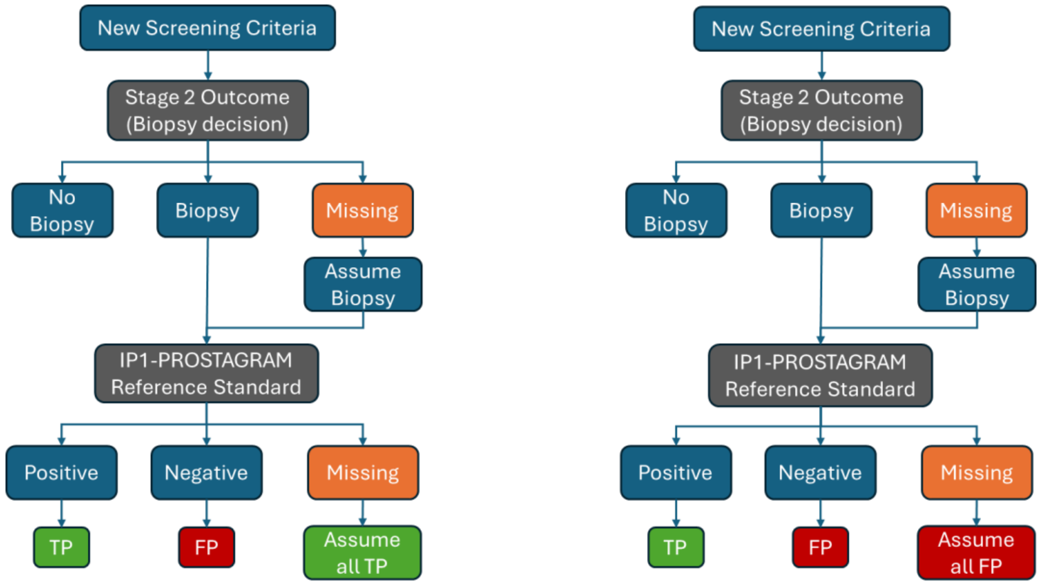

TP = true positive; FP = false positive

Figure S2 – Schematic representation which shows how missing data was accounted for when calculating the minimum and maximum positive predictive values (PPV) of different screening pathways during verification bias adjustment. To calculate the maximum PPV all missing data was assumed true positive (left flowchart) and to calculate the minimum PPV all missing data was assumed false positive (right flowchart).

Complete case analysis

Table S6 – Positive predictive value (PPV) of the different screening pathways (1-17) using complete case analysis (i.e. the unadjusted approach). PPV was only calculated in participants with known Stage 2 biopsy recommendations and known reference standard outcomes.

Screening Pathway	Non-diagnostic	Recommend Stage 2	Recommend biopsy	GG≥2 detection	PPV (n/N, 95% CI)
1	20	30	17	8	8/15, 53 (30, 75)
2	20	23	12	6	6/11, 55 (28, 79)
3	20	23	12	6	6/11, 55 (28, 79)
4	20	30	15	7	7/14, 50 (27, 73)
5	17	23	12	6	6/11, 55 (28, 79)
6	18	23	12	6	6/11, 55 (28, 79)
7	9	24	13	6	6/11, 55 (28, 79)
8	20	79	17	8	8/15, 53 (30, 75)
9	17	12	8	5	5/7, 71 (36, 92)
10	18	10	7	5	5/7, 71 (36, 92)
11	9	22	12	6	6/11, 55 (28,79)
12	17	71	16	8	8/15, 53 (30, 75)
13	18	34	13	6	6/11, 55 (28,79)
14	9	79	18	8	8/15, 53 (30,75)
15	15	14	8	5	5/7, 71 (36, 92)
16	5	72	17	8	8/15, 53 (30, 75)
17	6	31	13	6	6/11, 55 (28, 79)

GG≥2 = Grade Group ≥2 cancer i.e. clinically significant cancer; PPV = positive predictive value. Data are n or n/N, n (95% CI).

Minimum and Maximum PPV Estimates

Table S7 – Minimum and maximum positive predictive value (PPV) estimates of the different screening pathways (1-17). Participants with missing data at Stage 2 were all assumed to be recommended for biopsy. Participants with missing reference standard outcome were assumed to be true positive in the calculation for maximum PPV and false positive in the calculation for minimum PPV. A schematic representation is shown in Figure S2.

Screening Pathway	Recommend Stage 2	Recommend biopsy	GG≥2 detection	PPV minimum (n/N, 95% CI)	PPV maximum (n/N, 95% CI)
1	30	17	8-10	8/17, 47 (26, 69)	10/17, 59 (36, 78)
2	23	12	6-7	6/12, 50 (25, 75)	7/12, 58 (32, 81)
3	23	12	6-7	6/12, 50 (25, 75)	7/12, 58 (32, 81)
4	30	15	7-8	7/15, 47 (25, 50)	8/15, 53 (30, 75)
5	23	12	6-7	6/12, 50 (25, 75)	7/12, 58 (32, 81)
6	23	12	6-7	6/12, 50 (25, 75)	7/12, 58 (32, 81)
7	24	13	6-8	6/13, 46 (23, 71)	8/13, 62 (36, 82)
8	79	66	10-39	10/66, 15 (8, 26)	39/66, 59 (47, 70)
9	12	8	5-6	5/8, 62 (31, 86)	6/8, 75 (41, 93)
10	10	7	5	5/7, 71 (36, 92)	5/7, 71 (36, 92)
11	22	12	6-7	6/12, 50 (25,75)	7/12, 58 (32, 81)
12	71	59	9-33	9/59, 15 (8, 27)	33/59, 56 (43, 68)
13	34	22	7-15	7/22, 32 (16, 53)	15/22, 68 (47, 84)
14	79	66	10-39	10/66, 15 (8, 26)	39/66, 59 (47, 70)
15	14	10	5-8	5/10, 50 (24, 76)	8/10, 80 (49, 94)
16	72	60	10-34	10/60, 17 (9, 28)	34/60, 57 (44, 68)
17	31	20	7-13	7/20, 35 (18, 57)	13/20, 65 (43, 82)

GG≥2 = Grade Group ≥2 cancer i.e. clinically significant cancer; PPV = positive predictive value. Data are n or n/N, n (95% CI).

Multiple imputation

Table S8 – Positive predictive value (PPV) estimates of the different screening pathways (1-17) using standard and penalised (Firth's method[3]) logistic regression to account for verification bias.

Screening Pathway	Standard Logistic Regression			Penalised logistic regression (Firth's method)		
	PPV (95% CI)			PPV (95% CI)		
	Complete data at Stage 2	Missing data at Stage 2		Complete data at Stage 2	Missing data at Stage 2	
		Assume no biopsy	Assume biopsy		Assume no biopsy	Assume biopsy
1	47 (26, 69)			47 (26, 69)		
2	50 (25, 75)			50 (25, 75)		
3	50 (25, 75)			50 (25, 75)		
4	47 (25, 70)			47 (25, 70)		
5	50 (25, 75)			50 (25, 75)		
6	50 (25, 75)			50 (25, 75)		
7	46 (23, 71)			46 (23, 71)		
8		12 (6, 22)	16 (9, 27)		12 (6, 22)	16 (9, 27)
9	62 (31, 86)			62 (31, 86)		
10	71 (36, 92)			71 (36, 92)		
11	50 (25, 75)			50 (25, 75)		
12		14 (7, 25)	16 (9, 27)		14 (7, 25)	16 (9, 28)
13		27 (13, 48)	32 (17, 53)		27 (13, 48)	32 (17, 53)
14		12 (6, 22)	16 (9, 27)		12 (6, 22)	16 (9, 27)
15		50 (24, 76)	50 (24, 76)		50 (24, 76)	50 (24, 77)
16		13 (7, 24)	17 (10, 29)		13 (7, 24)	18 (10, 29)
17		30 (15, 52)	35 (18, 57)		30 (15, 52)	35 (18, 57)

PPV = positive predictive value. Data are n (95% CI).

Summary

A summary of the exploratory analyses is shown in Table S9. A comparable PPV to the original 'stage-gated' approach was observed for pathways without PSAd thresholds (pathway 2-4) and pathways consisting of two readers with a third reader as an arbitrator (pathway 5-7) or agreement between two readers at Stage 1 (pathway 9-11). In contrast, there was a wider variation in the PPV for pathways consisting of three readers with any one reader positive (pathway 8), two readers with either positive (pathway 12-14) and one reader only (pathway 15-17).

Table S9 – Summary table showing the positive predictive value (PPV) of different screening pathways adjusted for missing data/verification bias, using complete case analysis (i.e. the unadjusted approach), minimum/maximum estimates and multiple imputation using standard logistic regression.

**Screening Pathway**	**Statistical Approach for Calculating PPV (95% CI)**					
	Complete Case Analysis (unadjusted)	Minimum Estimate	Maximum Estimate	Multiple Imputation - complete Stage 2 data	Multiple Imputation – if missing Stage 2 data, participant assumed not to have biopsy	Multiple Imputation – if missing Stage 2 data, participant assumed to undergo biopsy
1	53 (30, 75)	47 (26, 69)	59 (36, 78)	47 (26, 69)		
2	55 (28, 79)	50 (25, 75)	58 (32, 81)	50 (25, 75)		
3	55 (28, 79)	50 (25, 75)	58 (32, 81)	50 (25, 75)		
4	50 (27, 73)	47 (25, 70)	53 (30, 75)	47 (25, 70)		
5	55 (28, 79)	50 (25, 75)	58 (32, 81)	50 (25, 75)		
6	55 (28, 79)	50 (25, 75)	58 (32, 81)	50 (25, 75)		
7	55 (28, 79)	46 (23, 71)	62 (36, 82)	46 (23, 71)		
8	53 (30, 75)	15 (8, 26)	59 (47, 70)		12 (6, 22)	16 (9, 27)
9	71 (36, 92)	62 (31, 86)	75 (41, 93)	62 (31, 86)		
10	71 (36, 92)	71 (36, 92)	71 (36, 92)	71 (36, 92)		
11	55 (28,79)	50 (25,75)	58 (32, 81)	50 (25, 75)		
12	53 (30, 75)	15 (8, 27)	56 (43, 68)		14 (7, 25)	16 (9, 27)
13	55 (28,79)	32 (16, 53)	68 (47, 84)		27 (13, 48)	32 (17, 53)
14	53 (30,75)	15 (8, 26)	59 (47, 70)		12 (6, 22)	16 (9, 27)
15	71 (36, 92)	50 (24, 76)	80 (49, 94)		50 (24, 76)	50 (24, 76)
16	53 (30, 75)	17 (9, 28)	57 (44, 68)		13 (7, 24)	17 (10, 29)
17	55 (28, 79)	35 (18, 57)	65 (43, 82)		30 (15, 52)	35 (18, 57)

PPV = positive predictive value. Data are % (95% CI).

Review of Incidental Findings in IP1-PROSTAGRAM and ReIMAGINE screening

Given that the 'stage-gated' reporting approach proposes that only limited screening sequences (axial T2-weighted and high *b*-value diffusion weighted imaging) are reviewed in screen-negative men, we aimed to assess whether this would correspond to a risk of missing incidental findings found on biparametric MRI in a prostate screening population.

Extra-prostatic incidental findings recorded in the ReIMAGINE Screening[4] (n=303) and IP1-PROSTAGRAM[1] (n=405) datasets were identified, and the MRI scans retrospectively reviewed to see if incidental findings were visible on the limited screening sequences. Incidental findings were divided into actionable (i.e. requiring further investigation/clinical correlation) and non-actionable findings.

All actionable extra-prostatic incidental findings requiring further investigation or clinical correlation were visible on just axial T2W and high *b*-value DWI sequences (Table S10). Most non-actionable extra-prostatic incidental findings were also visible (19 of 26, 73%). This suggests that the stage-gated approach has a low risk of potential harm from missed incidental findings.

Table S10 – Visibility of actionable (i.e. requiring further investigation/clinical correlation) and non-actionable incidental findings on limited screening sequences in IP1-PROSTAGRAM and ReIMAGINE Screening datasets.

Visible on limited screening sequences	Incidental Findings	
	Actionable (n)	Non-actionable (n)
**Yes**	5<br>(e.g. suspected bladder/rectal tumour, bone lesion)	19<br>(e.g. bladder diverticulum, inguinal hernia, hydrocele, uncomplicated diverticulosis)
**No**	0	7<br>(e.g. hydrocele, degenerative lumbosacral changes, inguinal hernia, uncomplicated diverticulosis)

n = number of scans

References

1. Eldred-Evans D, Burak P, Connor MJ, et al (2021) Population-Based Prostate Cancer Screening with Magnetic Resonance Imaging or Ultrasonography: The IP1-PROSTAGRAM Study. JAMA Oncol. https://doi.org/10.1001/jamaoncol.2020.7456

2. Day E, Eldred-Evans D, Prevost AT, Ahmed HU, Fiorentino F (2022) Adjusting for verification bias in diagnostic accuracy measures when comparing multiple screening tests - an application to the IP1-PROSTAGRAM study. BMC Med Res Methodol. https://doi.org/10.1186/s12874-021-01481-w

3. Firth D (1993) Bias Reduction of Maximum Likelihood Estimates. Biometrika. https://doi.org/10.2307/2336755

4. Moore CM, Frangou E, McCartan N, et al (2023) Prevalence of MRI lesions in men responding to a GP-led invitation for a prostate health check: a prospective cohort study. BMJ Oncology. https://doi.org/10.1136/bmjonc-2023-000057
